# AEO7 Surfactant as an Eco-Friendly Corrosion Inhibitor for Carbon Steel in HCl solution

**DOI:** 10.1038/s41598-018-37254-7

**Published:** 2019-02-19

**Authors:** Mostafa H. Sliem, Mohammed Afifi, A. Bahgat Radwan, Eman M. Fayyad, Mohamed F. Shibl, Fakiha El-Taib Heakal, Aboubakr M. Abdullah

**Affiliations:** 1 Center for Advanced Materials, Qatar University, Doha, P.O. Box 2713, Qatar; 2 Chemistry Department, Faculty of Science, Cairo University, Giza, 12613 Egypt; 3 Physical Chemistry Department, National Research Centre, Dokki, Cairo Egypt; 4 Chemistry Department, College of Arts and Sciences, Qatar University, Doha, P.O. Box 2713, Qatar

## Abstract

The impact of AEO7 surfactant on the corrosion inhibition of carbon steel (C-steel) in 0.5 M HCl solution at temperatures between 20 °C and 50 °C was elucidated using weight loss and different electrochemical techniques. The kinetics and thermodynamic parameters of the corrosion and inhibition processes were reported. The corrosion inhibition efficiency (*IE*%) improved as the concentration of AEO7 increased. In addition, a synergistic effect was observed when a concentration of 1 × 10^−3^ mol L^−1^ or higher of potassium iodide (KI) was added to 40 µmol L^−1^ of the AEO7 inhibitor where the corrosion *IE*% increased from 87.4% to 99.2%. Also, it was found that the adsorption of AEO7 surfactant on C-steel surface followed the Freundlich isotherm. Furthermore, electrochemical impedance spectroscopy (EIS) and potentiodynamic polarization measurements indicated that AEO7 was physically adsorbed on the steel surface. The surface topography was examined using an optical profilometer, an atomic force microscope (AFM), and a scanning electron-microscope (SEM) coupled with an energy dispersion X-ray (EDX) unit. Quantum chemical calculations based on the density functional theory were performed to understand the relationship between the corrosion *IE*% and the molecular structure of the AEO7 molecule.

## Introduction

Carbon steel (C-steel) is extensively utilized as a construction material in different industrial fields due to its high mechanical properties and low cost. It exists in most industrial infrastructures such as heat exchangers, reactors, drums, boilers and tubings^1,2^. In oil and gas industries, sulfuric and hydrochloric acids are commonly utilized as pickling acids for the oil wells acidification and the removal of undesirable scales such as rust or mill-scale from steel or any ferrous alloys at elevated temperatures. However, pickling acids are highly corrosive and their aggressiveness needs to be suppressed by adding suitable corrosion inhibitors in small ranges^3–5^. For these reasons, an intensive attention is paid for the corrosion of steel in different aggressive corrosion media^6–11^. Excellent corrosion inhibition properties are exhibited using inhibitors comprising electronegative atoms such as oxygen, sulfur, phosphorous and/or nitrogen as well as aromatic rings. Recently, eco-friendly biodegradable natural materials show perfect corrosion inhibition efficiencies as well as cost effectiveness. For instance, polymers, possessing polar functional groups such as hydroxyl, carboxylic and amino groups, manifest good corrosion inhibition in different corrosive media because they have a high affinity towards metallic surfaces^12–16^. For decades, surfactants are used in an extensive range of applications, from cleaning purposes such as shampoos, soap and detergents to industrial uses, namely, additives in coatings, concrete mixtures and paints. Moreover, researchers investigate the surfactant-flooding phenomenon in order to enhance the oil recovery. Furthermore, surfactants are utilized as corrosion inhibitors for several metals in the oil and gas production^17–20^. There are four types of surfactants: anionic, cationic, nonionic and amphoteric. The ethoxylated fatty alcohols surfactants are one of the most common nonionic surfactants that are used in the detergent industry and for an eco-friendly applications^21,22^. Nevertheless, their corrosion inhibition effectiveness is not yet well exploited especially in acidic environments^23,24^. For instance, Abdallah has investigated the effect of the numbers of ethylene oxide units of ethoxylated fatty alcohols, on the corrosion of zinc in 0.5 M HCl. It was found that to the corrosion inhibition increase with increasing the concentration and the number of ethylene oxide units per molecule. Inhibition was explained on the basis of adsorption of ethoxylated fatty alcohols molecules on the metal surface through their ethoxy groups^25^. Additionally, a novel eco-friendly Gemini surfactant is synthesized by Mobin *et al*., namely, Ethan-1,2-diyl bis(N,N-dimethyl-N-hexadecyl ammonium acetoxy) dichloride (16-E2-16). This mixed-type corrosion inhibitor considerably increases the corrosion inhibition efficiency (*IE*%) of mild steel to 98% in 1 M HCl at 60 °C^26^. Furthermore, three novel nonionic dithiol surfactants are synthesized based on 2-mercaptoacetic acid and polyethylene glycol with different molecular weights to obtain the di-mercaptoethanoate polyethylene glycol: SH600, SH1000 and SH1500. It is proved that the corrosion *IE*% increases with the molecular weight^27^. The combination of cetyl trimethyl ammonium bromide (CTAB) and indigo carmine significantly enhances the corrosion *IE*% of indigo carmine/CTAB for C-steel to 98.5% at 5 × 10^−5^ M in 0.5 M HCl^28^. In addition, a new green inhibitor extracted from Morus alba pendula leaves (MAPLE) is used. The corrosion *IE*% increases from 93% at a concentration of 0.4 g L^−1^ to 96% after adding1 mM of KI^29^. Moreover, ionic liquids e.g. tetra-*n*-butyl ammonium methioninate is used as a corrosion inhibitor owing to their electrostatic interaction with the iron surface (following Freundlich isotherm) with a maximum efficiency of 93.8% at 1.59 × 10^−3^ M of the inhibitor in 1 M HCl^30^. The aim of this work is to investigate AEO7 nonionic surfactant as an eco-friendly corrosion inhibitor for C-steel at ambient and elevated temperatures in 0.5 M HCl, which to the best of our knowledge, has not been previously reported literature. In addition, the adsorption isotherm and the thermodynamic parameters for the adsorption of the AEO7 inhibitor molecules on the C-steel surface are calculated. Furthermore, the synergistic effect of various concentrations of KI on the corrosion *IE*% of AEO7 for C-steel are studied. Moreover, quantum chemical parameters based on the density functional theory method are investigated. For example, the energies of the lowest unoccupied molecular orbitals (*E*_LUMO_) and the highest occupied molecular orbitals (*E*_HOMO_) as well as the energy gap (Δ*E*) are calculated. Moreover, the electron affinity (*A*), the ionization potential (*I*), the electronegativity *(X)*, the global softness (*δ)*, the dipole moment (μ) and the global hardness (γ) are figured out to provide a theoretical explanation for the corrosion inhibitive behavior of AEO7 and to understand the relation between the corrosion *IE*% and its molecular structure.

## Experimental

### Materials

**Figure 1:**
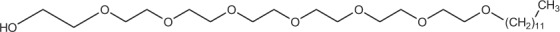
The chemical structure of the AEO7 molecule.

C-steel sheets were supplied by Qatar Steel Co., Ltd, Qatar. The chemical composition of C-steel is AISI 1020 alloy of iron and 0.2% carbon with up to 0.7% manganese, 0.65% silicon, and 0.65% copper in wt.%. Steel specimens were abraded to a 4000-grit finish using silicon papers, degreased in acetone, washed with deionized water, and dried in air. The 0.5 M HCl aggressive medium was prepared by diluting an analytical grade 37% HCl with deionized water. The AEO7 surfactant was obtained from Shanghai Dejun Technology Co., Ltd, China. The chemical structure of the surfactant is shown in Fig. 1. Different concentrations of AEO7 (10, 15, 17, and 20 ppm which correspond to 20, 30, 35, and 40 μmol L^−1^, respectively) were prepared in deionized water and used as inhibitors for C-steel corrosion in 0.5 M HCl. KI was purchased from Sigma-Aldrich Chemie Gmbh (Munich, Germany) to prepare solutions with and without AEO7 in which concentration were (1, 2.5, 5, 7.5, and 10) × 10^−3^ mol L^−1^.

### Weight loss measurements

The C-steel sheets with dimensions 2.5 × 2.0 × 0.2 cm^3^ were used for weight loss measurements. The weight loss measurements were performed in a 200 mL glass beaker containing 100 mL of 0.5 M HCl with and without adding different concentrations of AEO7 at room temperature. In order to investigate the effect of time on the rate of corrosion in the presence of the inhibitor, the performance was tested in a range from 30 min to 3 h. Afterwards, the specimens were removed and treated according to the method described in the ASTM G1-90 standard^31^. The corrosion rate (mpy) was computed based on Equation (1).

1 $${\rm{Corrosion}}\,{\rm{rate}}\,({\rm{mpy}})=\frac{534\,W}{\rho At}$$

where $W$ is mass loss in mg, *ρ* is the C-steel density in g cm^−3^, *A* is the surface area of the sample in cm^2^ and *t* is the time of the test in h.

The corrosion *IE*% and the surface coverage $\theta$ of the inhibitor used for the corrosion of C-steel were calculated as follows^15,32^,

2 $$IE \% =\theta \times 100=\frac{({W}^{0}-W)}{{W}^{0}}\times 100$$

where *W*^0^ and *W* are the average weight loss values without and with adding the inhibitor, respectively.

### Electrochemical measurements

Electrochemical measurements were performed in a three-electrode double-jacketed cell. The C-steel working electrodes were prepared according to the aforementioned procedures described in Section 2.1. It had an exposed area of 0.5 cm^2^. A graphite sheet with the same exposed area was used as a counter electrode and a saturated calomel electrode (SCE) acted as a reference electrode. The reference electrode was coupled with a Luggin capillary to minimize the *IR* potential drop. A Julabo F12 thermostat (GmbH, Seelach, Germany) was utilized to control the temperature of the solution as all electrochemical tests were carried out at various temperatures (20 °C, 30 °C, 40 °C, and 50 °C) in the presence and absence of different concentrations of the inhibitor. Before any electrochemical measurement, the C-steel electrode was dipped in the solution for 30 minutes to achieve a steady state condition. The EIS analyses were performed under an open circuit potential (OCP) condition within a frequency range of 0.1 Hz to 100 kHz with an AC amplitude of 10 mV using a GAMRY 3000 potentiostat/galvanostat/ZRA (Warminster, PA, USA). The potentiodynamic cathodic and anodic polarization curves varied from −250 mV to +250 mV versus open circuit potential (OCP) with a scan rate of 0.3 mV s^−1^. To ensure reproducibility, each test was repeated three times.

### Surface morphology

The surface of C-steel was examined using different characterization techniques. A Leica optical profilometer was utilized to explore the surface topography and surface roughness at a microscale. At the same time, an Asylum Research MFP-3D, atomic force microscope, AFM (Santa Barbara, CA, USA) was used to measure the surface roughness and surface topography in nanoscale and compared to the results that were obtained from the optical profilometer. The surface condition was checked using an FEI NOVA NANOSEM 450 high field emission scanning electron microscope, HFESEM, (Hillsboro, OR, USA) to document the corrosion of C-steel.

### Computational

A quantum chemical study was carried out using the density functional theory (DFT) method as implemented in the GAUSSIAN09 suit of programs^33^. Geometric optimization was performed using the B3LYP functional combined with the cc-pvdz basis set. The B3LYP functional (with Becke’s three- parameter functional (B3) and a mixture of HF with DFT exchange terms linked to the gradient-corrected correlation functional of Lee, Yang, and Parr (LYP)^34,35^) is known to produce a good estimate of molecular properties related to reactivity. The consistent-correlated polarized valence double zeta basis set (cc-pvdz) basis set^36,37^ became a state of the art for correlated calculations. The DFT, among other functions, seems promising for revealing changes in the electronic structure responsible for the inhibitory action of compounds on metal surfaces. The geometry of AEO7 was fully optimized using a gradient minimization technique. The optimum structure was characterized as having a zero-gradient norm. By diagonalizing the matrix of the second derivatives, positive harmonic vibrational frequencies were observed. The Gaussian 09 software was utilized to calculate the quantum chemical parameters necessary to explain the atomic and molecular interactions involving the compounds. GaussView 5.0 software was further applied in visualizing the electron density graphical isosurfaces and the quantum chemical parameters, which were calculated in the domain of Koopmans’ theory^38^ such as the energy of the highest occupied molecular orbital (*E*_HOMO_) and the energy of the lowest unoccupied molecular orbital (*E*_LUMO_). Moreover, the ionization potential (*I*) and the electron affinity (*A*) associated with the energies of HOMO and LUMO, respectively, where *I* = *−E*_HOMO_, *A* = *−E*_LUMO_ and the energy gap $({\rm{\Delta }}E={E}_{{\rm{HOMO}}}-{E}_{{\rm{LUMO}}})$, as well as the chemical potential (*μ*), the absolute hardness (χ), and the absolute softness were calculated.

The global hardness (*γ*) reflects the resistance to a charge transfer while the global softness (*δ*) describes the ability to receive electrons and were estimated by:

3 $$\gamma =-\,\frac{1}{2}({E}_{{\rm{Homo}}}-{E}_{{\rm{Lumo}}})$$

4 $${\rm{\delta }}=\frac{1}{\gamma }\cong -\,\frac{2}{{E}_{{\rm{HOMO}}}-{E}_{{\rm{LUMO}}}}$$

Electronegativity (*X*) is the power of an atom to attract electrons towards itself and was calculated using Koopmans’ theory

5 $$X=-\,\frac{1}{2}({E}_{{\rm{Homo}}}+{E}_{{\rm{Lumo}}})$$

## Results and Discussion

### Weight loss measurements

#### Effect of inhibitor concentration on corrosion of C-steel

**Figure 2:**
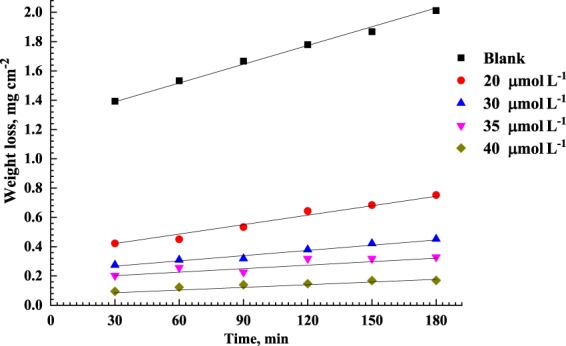
Weight loss transients for C-steel upon immersion in 0.5 M HCl in the presence and absence of 20, 30, 35, and 40 μmol L^−1^ of AEO7 at 25 °C.

Figure 2 shows the weight loss transients for C-steel upon immersion in 0.5 M HCl in the presence and absence of 20, 30, 35, and 40 μmol L^−1^ of AEO7 at 25 °C. It reveals that the higher the concentration of AEO7 is, the lower the weight loss we measure i.e. the higher the corrosion *IE*% we calculate. The result can be attributed to the ability of AEO7 to be adsorbed on the surface of C-steel because of the existence of seven oxygen atoms arranged in a kind of chain sequence in AEO7 which helps the 3d orbitals of an iron atom to interact with the lone pairs of electrons from the oxygen atoms^39–41^. This, consequently, increases the corrosion *IE*%. In addition, the high molecular weight of AEO7, because of the presence of a long chain of hydrophobic ethylene groups, increases the corrosion *IE*%. Therefore, in the presence of AEO7, the corrosion rate is an indicator for the number of free corrosion sites remaining after effectively blocking the other sites by the adsorbed inhibitor molecule^25,42^.

#### Effect of immersion time on corrosion of C-steel

Assuming that corrosion takes place only at the free sites while neglecting the corrosion rates of the covered sites, the corrosion *IE*% can be calculated by Equation 2. Table 1 shows the results for corrosion *IE*% of different concentrations of AEO7 on C-steel after various immersion times at 25 °C. The increase in the corrosion rate with time is attributed to the entanglement of the polymer chains with each other with time which lowers the overall entropy surface energy meanwhile exposes more active sites for corrosion^43,44^.

**Table 1:** Corrosion *IE*% of AEO7 for the corrosion of C-steel in 0.5 M HCl after different exposure times at 25 °C.

*C*_inh_ (µmol. L^−1^)	Corrosion *IE*%					
	30 min	60 min	90 min	120 min	150 min	180 min
Blank	—	—	—	—	—	—
20	69.7	70.6	67.1	63.8	63.4	62.6
30	80.3	79.8	80.9	78.6	77.4	77.5
35	85.4	83.2	86.4	82.1	82.9	83.7
40	93.2	91.9	91.6	91.7	91.0	91.6

### EIS measurements

**Figure 3:**
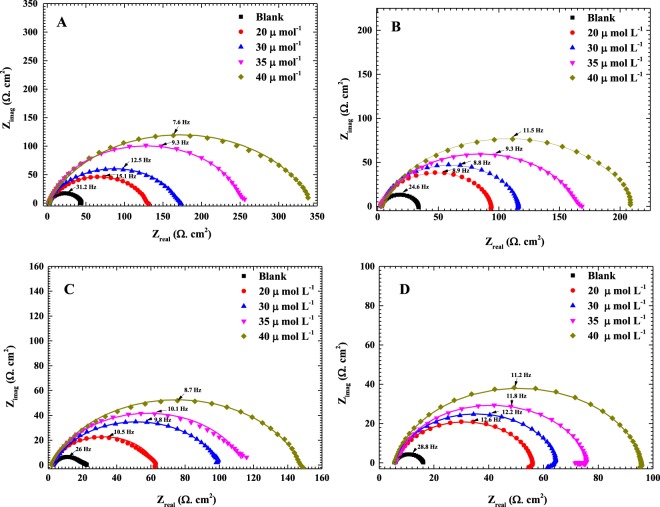
Measured EIS Nyquist plots (dotted) and their fitted curves (solid lines) for C-steel in 0.5 M HCl solution in the presence of 0, 20, 30, 35 and 40 µmol L^−1^ of AEO7 corrosion inhibitor at (**A**) 20, (**B**) 30, (**C**) 40 and (**D**) 50 °C. The fitting is done using the equivalent circuit shown in Fig. 4.

Figure 3 shows the EIS Nyquist plots for C-steel in 0.5 M HCl solution in the absence and presence of 20, 30, 35 and 40 µmol L^−1^ of the AEO7 corrosion inhibitor. Clearly, the diameters of the semicircles in the presence of AEO7 inhibitor are larger compared to the blank one. It is also lucid that the diameters of the semicircles decrease as the temperature increases at the same concentration of the AEO7 inhibitor. In addition, the depressed capacitive loops at low frequency in all the Nyquist plots refer to a charge transfer-controlled mechanism for the corrosion of C-steel in the 0.5 M HCl solution. The deviation of the capacitive loop from a complete semi-circle can be referred to the heterogeneity and microroughness of the working electrode surface^45,46^.

**Figure 4:**
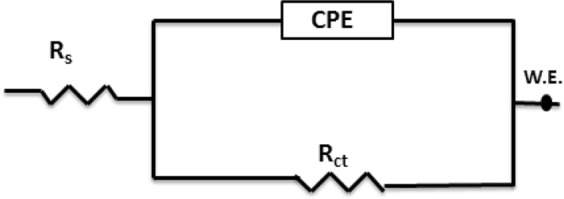
An equivalent electrical circuit used for the analysis of EIS measured data.

Figure S1 (Supporting Information) depicts the Bode plots and phase angle curves for the same EIS data represented by the Nyquist plots shown in Fig. 3. In contrast to the effect of increasing the temperature, raising the AEO7 concentration increases the phase angles and shifts their peaks to lower frequencies^47–50^. It can be seen that the phase angles for the inhibited cases are much higher than the uninhibited ones. The increased values of the phase angle for the inhibited specimens indicated that the surface becomes smoother owing to the inhibitor adherence to the metallic surface and the formation of a protective film of inhibitors over the metallic surface. The broadening and the shift to lower frequencies of the single maximum in the Bode plots further supports the protective film formation by inhibitor molecules^51,52^. Figure 4 shows a one-time constant equivalent electrical circuit (EC) that is used for fitting the measured EIS data of a uniformly corroding electrodes in an electrolyte^53,54^. The EC is composed of an uncompensated solution resistance (*R*_s_), a charge transfer resistance (*R*_ct_) and a constant phase element (CPE) that replaces the capacitive element to obtain a more accurate fit. CPE is used for a non-ideal double-layer capacitor. The non-ideal behavior of a double layer can be attributed to many reasons; e.g, surface roughness and a non-uniform (i) surface coverage, (ii) corrosion rate and/or (iii) current distribution.

The impedance of the CPE is expressed by Equation 6 ^55^:

6 $${Z}_{Q}=[{Y}_{0}^{-1}{(j\omega )}^{-n}]$$

where *Z*_Q_ is CPE impedance (Ω cm^−2^), *Y*_0_ is the CPE constant in µs^n^ ohm^−1^ cm^−2^, *j* = (−1)^1/2^, *ω* is the angular frequency in rad s^−1^ and the value of *n* ranges between 0 and 1. When n = 1, the CPE becomes equivalent to an ideal capacitor, and when *n* = 0, the CPE becomes equivalent to a resistor. The double layer capacitance (*C*_dl_) can be calculated using the following equation^56^:

7 $${C}_{{\rm{dl}}}=\frac{{({Y}_{0}{R}_{ct})}^{1/n}}{{R}_{ct}}={Y}_{0}{\omega }^{(n-1)}$$

The surface coverage (*θ*) is estimated using the following equation:

8 $$\theta =\frac{{R}_{{\rm{ct}}1}-{R}_{{\rm{ct}}2}}{{R}_{{\rm{ct}}1}}$$

where *R*_ct1_ and *R*_ct2_ are the charge-transfer resistances in the presence and absence of the inhibitor, respectively. Moreover, the corrosion *IE*% is calculated using Equation 2^55,56^. All EIS parameters obtained from the Nyquist and Bode graphs are summarized in Table 2.

**Table 2:** EIS parameters and corrosion inhibition efficiencies obtained from fitting impedance spectra of C-steel in 0.5 M HCl at different concentrations of AEO7 at 20, 30, 40 and 50 °C using the electrical equivalent circuit shown in Fig. 4.

*T* (°C)	*C*_inh_ µmol L^−1^	*R*_ct_, Ω cm^2^	*CPE*			$\theta$	*Corrosion IE*%
			*Y*_0_ µs^n^ ohm^−1^ cm^−2^	*C*_dl_, µF	*n*		
20	Blank	43.1	257.0	119.7	0.855	—	—
	20	128.2	211.7	82.57	0.793	0.664	66.4
	30	169.8	194.5	76.85	0.786	0.746	74.6
	35	253.0	161.4	69.31	0.791	0.830	83.0
	40	341.6	105.95	59.49	0.852	0.874	87.4
30	Blank	30.8	381.8	218.7	0.889	—	—
	20	90.42	314.1	202.2	0.890	0.660	66.0
	30	114.2	260.4	156.8	0.874	0.730	73.0
	35	164.5	236.8	103.6	0.797	0.813	81.23
	40	211.5	149.2	64.25	0.804	0.854	85.4
40	Blank	24.27	425.0	252.7	0.898	—	—
	20	64.8	405.7	231.0	0.866	0.625	62.5
	30	89.0	390.3	185.7	0.819	0.727	72.7
	35	112.2	377.0	141.9	0.764	0.786	78.6
	40	145.8	331.4	124.6	0.756	0.833	83.3
50	Blank	18.5	606.5	295.6	0.862	—	—
	20	49.27	554.2	254.1	0.822	0.622	62.2
	30	59.32	543.7	217.4	0.789	0.688	68.8
	35	69.72	515.7	187.7	0.890	0.734	73.4
	40	90.42	492.8	155.0	0.729	0.795	79.5

It can be noticed that the higher the concentration of the AEO7 inhibitor is, the larger the charge transfer resistance we measure which is typically opposite to the behavior of the *C*_dl_ which decreases with increasing the AEO7 concentration or decreasing the temperature^57,58^. For instance, comparing the blank solution with the one containing 20 μmol L^−1^ of AEO7 at 20 °C, the *R*_ct_ increases from 43 Ω cm^2^ to 128 Ω cm^2^, and *C*_dl_ decreases from 119.75 µF to 82.57 µF (*IE*% is 66.4%). Meanwhile, increasing the inhibitor concentration to 40 μmol L^−1^ increases the *R*_ct_ value to 341.6 Ω cm^2^, and the *C*_dl_ decreases to 59.51 µF with an *IE*% of 87.6%. This observation can be explained using the Helmholtz equation^55^.

9 $${\delta }_{ads}=\frac{{{\rm{\varepsilon }}{\rm{\varepsilon }}}_{o}A}{{C}_{{\rm{dl}}}}$$

where *δ*_ads_ is the thickness of the adsorbed layer of the AEO7 corrosion inhibitor, ε_o_ is the vacuum permittivity, ε is the local relative dielectric constant and *A* is the area of C-steel electrode. This equation shows that *C*_dl_ is inversely proportional to *δ*_ads_ i.e. the decrease of the *C*_dl_ value is attributed to the growth of the adsorbed film of AEO7 corrosion inhibitor as its concentration increases in solution. As the protective film grows, the charge transfer becomes more sluggish as indicated by the high *R*_ct_ and corrosion *IE*% values. Increasing the temperature will decrease the corrosion *IE*% because the desorption rate of the inhibitor molecules from the C-steel surface increases which leads to an increase in the dissolution rate of the steel^59^.

In general, the higher the inhibitor concentration is, the less positive the *n* values are. This indicates that the behavior of the constant phase element is becoming farther from the ideal capacitor as the AEO7 concentration increases^60,61^.

### Potentiodynamic polarization measurements

**Figure 5:**
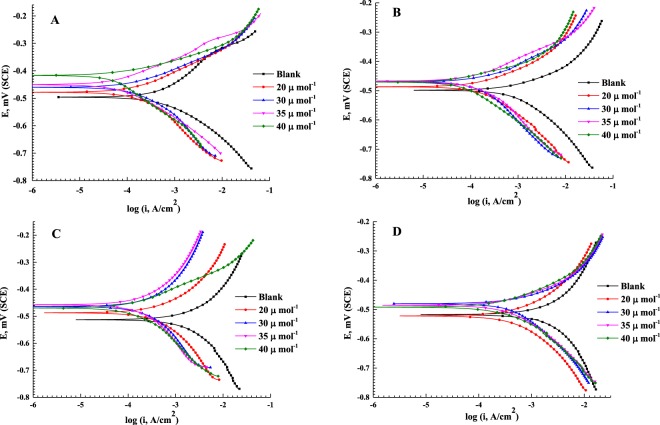
Potentiodynamic polarization curves for C-steel at a scan rate of 0.3 mV s^−1^ in 0.5 M HCl in the presence of 0, 20, 30, 35, 40 μmol L^−1^ of AEO7 corrosion inhibitor at (**A**) 20, (**B**) 30, (**C**) 40 and (**D**) 50 °C.

Figure 5 shows the potentiodynamic polarization curves of the C-steel in a 0.5 M HCl solution at a scan rate of 0.3 mV s^−1^ in the presence of 0, 20, 30, 35 and 40 µmol L^−1^ of AEO7 corrosion inhibitor at (A) 20, (B) 30, (C) 40 and (D) 50 °C. The electrochemical kinetic parameters such as the corrosion free potential (*E*_corr_), corrosion current density (*i*_corr_), the polarization resistance (*R*_p_) and the cathodic and anodic Tafel slopes (*ß*_c_ and *ß*_a_, respectively) which are obtained by the Tafel extrapolation method are listed in Table 3. The corrosion rates are calculated using Equation 10 assuming that the whole surface of the C-steel is attacked by the aggressive media, and no localized corrosion is detected^62,63^.

10 $${\rm{corrosion}}\,{\rm{rate}}(\mathrm{mm}/\mathrm{year})=\frac{{i}_{{\rm{corr}}}\,\ast \,10\,\ast \,M\,\ast \,3.15\,\ast \,{10}^{7}}{F\,\ast \,n\,\ast \,d}$$

**Table 3:** Potentiodynamic polarization parameters (derived from Fig. 5) for C-steel in 0.5 M HCl in the presence of 0, 20, 30, 35, 40 μmol L^−1^ of AEO7 corrosion inhibitor at different temperatures.

T (°C)	*C*_inh_ µmol L^−1^	^−*E*^_corr_ (mV) SCE	*i*_corr_ (µA cm^−2^)	*β*_a_, mV decade^−1^	*β*_c_, mV decade^−1^	*R*_*p*,_ Ω cm^2^	*CR, mm year* ^−1^	$\theta$	*IE* %
20	Blank	496.0	624	152.8	164.8	55.2	7.24	—	—
	20	478.0	194	97.2	157.9	130.1	2.25	0.68	68.91
	30	460.0	130	87.8	148.6	199.9	1.5	0.80	80.40
	35	458.0	71.5	68.1	123.6	266.9	0.83	0.88	88.54
	40	417.0	45.5	40.1	112.6	282.5	0.52	0.92	92.70
30	Blank	499.0	1056	202.2	192.8	40.6	12.26	—	—
	20	487.0	353.68	122	173.3	88.01	4.1	0.66	66.5
	30	471.0	250.9	107.5	162.9	127.9	2.91	0.79	79.3
	35	467.0	150.4	85.5	148.5	156.8	1.74	0.85	85.75
	40	470.0	95.8	56.8	129	178.9	1.11	0.90	90.92
40	Blank	513.0	1640	249.5	328.4	37.5	19.04	—	—
	20	487.0	567	211.2	272.1	91.1	6.58	0.65	65.42
	30	464.0	419	183.5	250.5	122.1	4.86	0.77	77.45
	35	457.0	277	149.5	225.7	141.1	3.21	0.83	83.10
	40	469.0	166	93.8	143.9	148.7	1.92	0.89	89.87
50	Blank	518.0	2440	423	352	34.2	28.34	—	—
	20	522.0	896	281	323.9	73.0	10.4	0.63	63.27
	30	480.0	650	183.1	285.3	80.8	7.54	0.75	75.36
	35	486.0	425	98.1	260.6	68.8	4.93	0.81	81.70
	40	492.0	301	44.9	235.3	54.4	3.49	0.78	87.66

3.15 * 10^7^ is the number of seconds in one year and 10 is a factor to change cm to mm. *M* is the atomic weight of iron (g mol^−1^) while *n* is the number of transferred electrons per metal atom, *F* is Faraday’s constant (96497 C mol^−1^), *A* is the exposed area of the electrode, *d* is the density of iron, and *i*_corr_ is the corrosion current density in A cm^−2^.

The polarization resistance (*R*_*p*_), can be calculated using the Stern–Geary equation^55^.

11 $${R}_{{\rm{p}}}=\frac{{\beta }_{{\rm{c}}}{\beta }_{{\rm{a}}}}{2.303\,{i}_{{\rm{corr}}}({\beta }_{{\rm{c}}}+{\beta }_{{\rm{a}}})}$$

The values of surface coverage ($\theta$) are calculated using the following equation:

12 $$\theta =\frac{{i}_{{\rm{corr}}}^{\text{'}}-{i}_{{\rm{corr}}}}{{i}_{{\rm{corr}}}^{\text{'}}}$$

where ${i}_{{\rm{corr}}}^{\text{'}}$ and ${i}_{{\rm{corr}}}$ are the corrosion current densities in the absence and presence of the AEO7 corrosion inhibitor. In addition, the corrosion *IE*% is calculated using Equation 2^55^.

It is worth noting that the presence of AEO7 corrosion inhibitor decreases the anodic and cathodic current densities and the corrosion potentials are shifted towards the nobler direction compared to the blank. This is a typical behavior for the mixed-type corrosion inhibitors^64^. It is reported that an inhibitor to be considered as cathodic or anodic one requires a shift of ±85 mV in the *E*_corr_ between the uninhibited and inhibited systems, otherwise it is classified as a mixed type one^44,65^. The *E*_corr_ values, in Table 3, confirm that AEO7 is of the mixed type category. The higher the concentration of the AEO7 is, the lower the corrosion rates are at any temperature. This can be explained by the increase in the local electron density at the steel surface by which the affinity of C-steel towards the adsorption of negatively charged Cl^−^ ions decreases and consequently the corrosion rate is lowered. On the other hand, raising the temperature increases the corrosion rate at the same concentration of the AEO7 due to the increase in the desorption rate of the corrosion inhibitor from the C-steel surface^66^. It is worth mentioning that the polarization data shown in Fig. 5 confirm the EIS ones in Figs 3 and S1.

### Synergism Analysis

**Figure 6:**
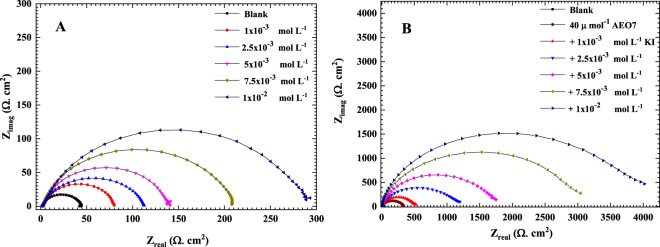
EIS Nyquist plots for C-steel in 0.5 M HCl at 20  C with 0, 1, 2.5, 5, 7.5, 10 × 10^−3^ M of KI in the (**A**) absence and (**B**) presence of 40 µmol L^−1^ of AEO7. Solid lines are the fittings that are done using the equivalent circuit shown in Fig. 4 while the symbols are the measured data.

Figure 6 illustrates the EIS Nyquist plots of C-steel in 0.5 M HCl at 20 °C in the presence of (0, 1, 2.5, 5, 7.5, 10) × 10^−3^ mol L^−1^ KI (A) without and (B) with 40 µmol L^−1^ of AEO7 corrosion inhibitor. It is clear that the diameters of the semicircles increase as the concentration of KI increases (compare the plots in Fig. 6B). The maximum efficiency of AEO7 corrosion inhibitor increases from 87.4 in the absence of KI to 99.2% in the presence of 1 × 10^−2^ mol L^−1^ of it. It is reported that the addition of halide ions to the corrosion inhibitors can, synergistically, enhance the corrosion inhibition efficiencies in aggressive media^28,57,67–69^. The iodide ions compete with the chloride ions for getting adsorbed on the steel surface then the AEO7 corrosion inhibitor molecules are attracted to the adsorbed iodide ions electrostatically as iodide ions increase the absorbability of the AEO7 by forming interconnecting bridges between the C-steel surface and the inhibitor molecules^67,68^. Tables 4 and S1 summarize the results of the corrosion inhibition measurements and calculations for C-steel in 0.5 M HCl in the presence of different concentrations of KI with and without 40 µmol L^−1^ of AEO7.

**Table 4:** The electrochemical parameters and corrosion inhibition efficiencies obtained from the measured impedance spectra of C-steel in 0.5 M HCl at different concentrations of KI with 40 µmol L^−1^ of AEO7 at room temperature.

*C*_inh_, mol L^−1^	*R*_ct_, Ω cm^2^	*CPE*			$\theta$	*Corrosion IE*%
		*Y*_0_ × 10^−6^ s^n^ ohm^−1^ cm^−2^	*C*_*dl*_, µF	*n*		
Blank	43.1	257.0	119.7	0.855	—	—
AEO7	341.6	105.95	67.91	0.852	0.87	87.4
1 × 10^−3^	493	63.98	42.10	0.892	0.913	91.3
2.5 × 10^−3^	1196	51.08	37.31	0899	0.963	96.3
5 × 10^−3^	1973	52.45	34.81	0.847	0.978	97.8
7.5 × 10^−3^	3250	48.70	32.58	0.821	0.986	98.6
1 × 10^−2^	4351	24.61	9.73	0.719	0.992	99.2

To determine the synergistic effect of KI, the synergism parameter S_1_ is calculated using Aramaki and Hackerman’s Equation^70^:

13 $${S}_{1}=\frac{1-{\theta }_{1+2}}{1-{\theta }_{1+2}^{\text{'}}}$$

where *θ*_1+2_ = (*θ*_1_ + *θ*_2_) − (*θ*_1_ × *θ*_2_), *θ*′_1+2_ represents the measured surface coverage for a certain concentration of KI in combination with a 40 μmol L^−1^ of AEO7, *θ*_1_ is the surface coverage of KI at a certain concentration and *θ*_2_ is the surface coverage of 40 μmol L^−1^ AEO7. The synergistic effect of KI with other inhibitors is common. However, in this research the interaction between KI and AEO7 is changing according to the concentration of iodide ions as seen in Table 5. Three conditions are reported with altering the KI concentration leading to different *S*_1_ values. If *S*_1_ values are more than unity, then the synergistic effect of KI is proved. However, if *S*_1_ values approach unity, it means that there is no interaction between potassium iodide and the AEO7 molecule. If *S*_1_ values are less than unity, an antagonistic behavior is expected which is a characteristics of a competitive adsorption process^69,71,72^. The synergism is originated from the double role that iodide ions play. The protective iodide ions compete with the chloride corrosive ones for the adsorption on the surface of the metal and at the same time they increase the adsorbability of AEO7 molecules on the C-steel surfaces^43,44^. Table 5 shows the values of *S*_1_ that are calculated after the addition of different concentrations of KI to a 0.5 M HCl solution with 40 μmol L^−1^ of AEO7. It is evident that the values are greater than unity for all KI concentrations that are higher than 1 × 10^−3^ M which proves the synergism between KI and AEO7.

**Table 5:** Calculated synergistic parameter *S*_1_ for different concentration of KI for C-steel in 0.5 M HCl in the presence of 40 μmol L^−1^ of AEO7 corrosion inhibitor at 20 °C.

*C*, mol L^−1^ (KI)	*S*_1_
1 × 10^−3^	0.672
2.5 × 10^−3^	1.230
5 × 10^−3^	1.773
7.5 × 10^−3^	1.857
1 × 10^−2^	1.773

### Inhibitor Adsorption and thermodynamic analysis

The equilibrium constants for the adsorption of AEO7 on the C-steel surface in a 0.5 M HCl solution at different temperatures can be derived from the proper adsorption isotherm. Freundlich adsorption isotherm shows the best fitting for the surface coverage of AEO7 on C-steel with its concentration in solution^73^. This isotherm can be summarized by Equations 14 and 15, as given below:

14 $$\theta ={K}_{{\rm{ads}}}{C}^{n}$$

15 $$\mathrm{log}\,\theta =\,\mathrm{log}\,{K}_{{\rm{ads}}}+2.303\,n\,\mathrm{log}\,C$$

where *C* is the concentration of the corrosion inhibitor, ${K}_{{\rm{ads}}}$ is the equilibrium constant for the adsorption process and *θ* is the surface coverage (obtained from the potentiodynamic polarization analysis shown in Table 3) while *n* is a function in the strength of adsorption.

**Figure 7:**
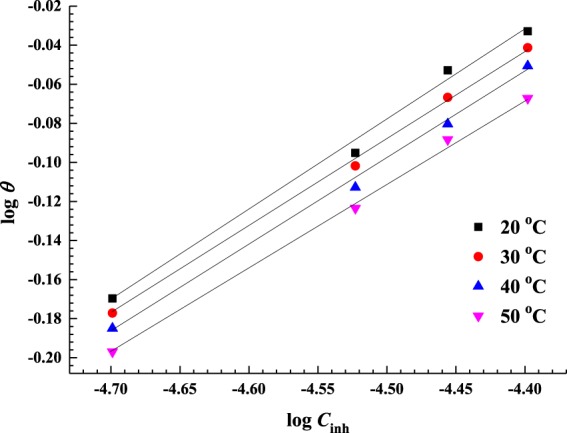
Freundlich isotherm plots for the adsorption of AEO7 on C-steel in 0.5 M HCl at different temperatures.

**Figure 8:**
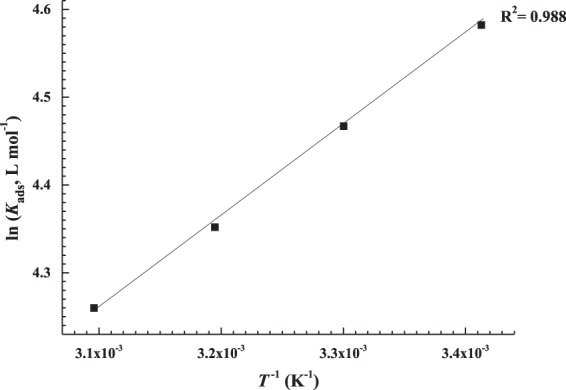
Van’t Hoff plot for the AEO7 corrosion inhibitor on C-steel surface in 0.5 M HCl at different temperatures.

Equation 15 is used to create Fig. 7 at various temperatures. *R*^2^ values are close to unity confirming the properness of using the Freundlich isotherm^30,74^. From the intercepts of the graphs, *K*_ads_ values are computed. *K*_ads_ constants are also used to calculate the values of the standard Gibbs free energy change of adsorption Δ*G°*_ads_ according to the following Equation^75,76^

16 $${\rm{\Delta }}{G}_{{\rm{a}}{\rm{d}}{\rm{s}}}^{{\rm{o}}}=-\,{\rm{R}}T\,{\rm{l}}{\rm{n}}(55.5\,{K}_{ads})$$

where R is the universal gas constant in J mol^−1^ K^−1^, *T* is the temperature in K and 55.5 is the number of moles of water per liter, according to Bouklah *et al*.^77^. Calculated values of *R*^2^, *K*_ads_ and Δ*G°*_ads_ are listed in Table 6. In addition, the enthalpy change of adsorption, ln *K*_ads_, was plotted versus 1/*T*, which resulted in a straight line, as shown in Fig. 8, following the Van’t Hoff equation.

**Table 6:** Thermodynamic parameters derived and calculated based on Freundlich adsorption isotherms and polarization measurements.

Temperature, K	*K*_ads_	*R* ^2^	*∆G*°_ads_ (kJ mol^−1^)	*∆H*°_ads_ (kJ mol^−1^)	*∆S*°_ads_ (J. mol^−1^ K^−1^)
293.15	97.723	0.995	−20.94	−8.90	41.00
303.15	87.096	0.998	−21.37	−8.90	41.15
313.15	77.624	0.990	−21.78	−8.90	41.44
323.15	70.795	0.994	−22.22	−8.90	41.30

There are different types of adsorption that represent the interaction between the metal surface and the corrosion inhibitor molecule e.g. physical, chemical or chemi/physisorption. From the results in Table 6, ${\rm{\Delta }}{G}_{{\rm{ads}}}^{{\rm{o}}}$ are negative and the absolute values are in the range of 20.94–22.22 kJ mol^−1^. Generally, if ${\rm{\Delta }}{G}_{{\rm{ads}}}^{{\rm{o}}}$ ≤ −20 kJ mol^−1^, it is linked to physisorption while if it is ≥−40 kJ mol^−1^, then it is inclined to chemisorption^7,60^. In recent reports, ${\rm{\Delta }}{G}_{{\rm{ads}}}^{{\rm{o}}}$ values in the range from −28 to −38 kJ/mol are interpreted as mixed adsorption (i.e both physisorption and chemisorption)^61,63^. The ${\rm{\Delta }}{G}_{{\rm{ads}}}^{{\rm{o}}}$ values obtained in the present investigation fall close to the category of the physisorption type of adsorption.

*K*_ads_ values are often used to describe the strength of the bond between adsorbent and adsorbate^63^. It can be seen in Table 6 that increasing the temperature decreases the *K*_ads_ as the bonds between C-steel and AEO7 molecules are weakened.

${\rm{\Delta }}{G}_{{\rm{ads}}}^{{\rm{o}}}$ is connected to ${\rm{\Delta }}{H}_{{\rm{ads}}}^{{\rm{o}}}$ (standard enthalpy) and ${\rm{\Delta }}{S}_{{\rm{ads}}}^{{\rm{o}}}$ (standard entropy) of adsorption according to Equation 17:

17 $${\rm{\Delta }}{G}_{{\rm{a}}{\rm{d}}{\rm{s}}}^{{}^{\circ }}={\rm{\Delta }}{H}_{{\rm{a}}{\rm{d}}{\rm{s}}}^{{}^{\circ }}\,{\textstyle \text{-}}\,T{\rm{\Delta }}{S}_{{\rm{a}}{\rm{d}}{\rm{s}}}^{{}^{\circ }}$$

Combining and rearranging Equations 16 and 17 lead to the Van’t Hoff Equation below (Equation 18)

18 $$\mathrm{ln}\,{K}_{{\rm{ads}}}=\frac{-{\rm{\Delta }}{H}_{{\rm{ads}}}^{\circ }}{{\rm{R}}T}+\frac{{\rm{\Delta }}{S}_{{\rm{ads}}}^{\circ }}{{\rm{R}}}-\,\mathrm{ln}\,55.5\,$$

${\rm{\Delta }}{H}_{{\rm{ads}}}^{^\circ }$ is obtained from the slope of the Van’t Hoff plot and ${\rm{\Delta }}{S}_{{\rm{a}}{\rm{d}}{\rm{s}}}^{{}^{\circ }}$ is obtained from Equation 17 and listed in Table 6.

The ${\rm{\Delta }}{H}_{{\rm{ads}}}^{^\circ }$ values can provide a vital information pertaining to the adsorption mechanism of an inhibitor. In the present study, ${\rm{\Delta }}{H}_{{\rm{ads}}}^{^\circ }$ value is negative implying exothermic adsorption. Similar results are reported by Esmaelili *et al*.^78^. The negative value of ${\rm{\Delta }}{H}_{{\rm{ads}}}^{^\circ }$ value is confirmed by the decrease in the corrosion *IE*% with increasing the temperature. On the other hand, the calculated values of ${\rm{\Delta }}{S}_{{\rm{ads}}}^{^\circ }$ are positive which is attributed to the increase in the solvent energy in addition to the increase in entropy due to H_2_O desorption^79^. Noor *et al*. also referred the positive ${\rm{\Delta }}{S}_{{\rm{ads}}}^{^\circ }$ values to the substitution of several water molecules by a single inhibitor molecule^80^.

### Analysis of Corrosion Kinetics

The effect of temperature on the dissolution of C-steel specimen in a 0.5 M HCl solution in the presence and absence of different concentrations of AEO7 is examined between 20–50 °C. Consequently, the corrosion kinetics parameters, namely, *E*_a_ (activation energy), ΔH* (activation enthalpy), and ΔS* (activation entropy) are calculated from the Arrhenius and transition state equations,

*E*_a_ can be calculated using Arrhenius Equation^81,82^:

19 $$\mathrm{log}\,CR=\,\mathrm{log}\,A-\frac{{E}_{{\rm{a}}}}{2.303\,RT}$$

where *CR* stands for the corrosion rate at a temperature *(T)* which is expressed by the corrosion current density (*i*), *E*_a_ represents the apparent activation energy and *A* is the Arrhenius constant that depends on the metal type and electrolyte. R is the universal gas constant (8.314 J mol^−1^ K^−1^).

**Figure 9:**
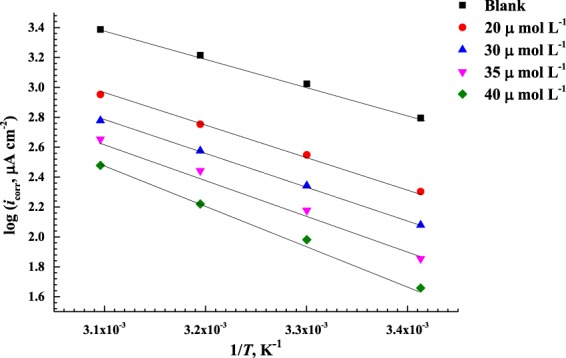
Arrhenius plots for the corrosion current densities (log *i*) versus 1/*T* at different concentrations of the AEO7 corrosion inhibitor in 0.5 M HCl.

Figure 9 shows the relation between log *i* and 1/*T* for C-steel in 0.5 M HCl solution with and without AEO7. From the slopes, *E*a can be calculated for each concentration.

**Figure 10:**
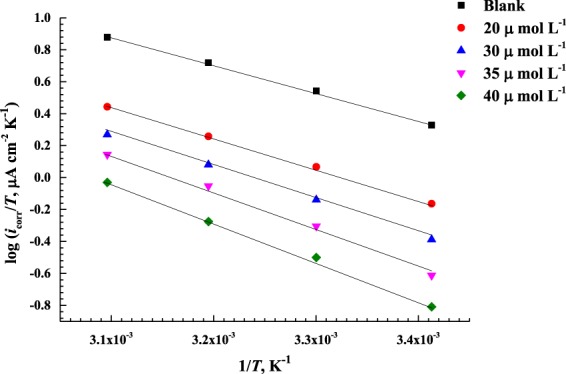
Transition-state plots of log *(i*/*T)* versus 1/*T* for C-steel in 0.5 M HCl with and without different concentrations of AEO7 corrosion inhibitor.

The activation enthalpy (∆H*) and activation entropy (∆S*), for the corrosion of C-steel in a 0.5 M HCl with and without AEO7 are calculated from the transition-state equation, as shown by Shuklar *et al*.^83^.

20 $$CR=\frac{{\rm{R}}T}{Nh}{e}^{\frac{{\rm{\Delta }}{S}^{\ast }}{R}}{e}^{\frac{-{\rm{\Delta }}{H}^{\ast }}{{\rm{R}}T}}$$

where *CR* is the rate of corrosion which is expressed by the current density, R is the universal gas constant in J mol^−1^. K^−1^, *T* is the temperature in K and *h* is the Planck’s constant. To obtain ∆H^*^ and ∆S^*^, log *i/T* versus 1/*T* are plotted at different concentrations of AEO7 corrosion inhibitor as displayed in Fig. 10.

ΔH* values are computed from the slopes of the plots while ΔS* values are calculated from the intercept of the plots in Fig. 10. The computed values of *E*a, ΔH* and ΔS* are presented in Table 7. Clearly, it is seen that ΔH* values in the presence of AEO7 corrosion inhibitor are smaller than those for AEO7-free HCl solutions. In addition, raising the concentration of AEO7 increases *E*a and ΔS* values. This trend supports the physisorption mechanism which is proposed for the adsorption of AEO7 molecules on the C-steel surface in a 0.5 M HCl solution.

**Table 7:** Activation energy (*E*_a_), activation enthalpy (∆H*) and activation entropy (∆S*) for C-steel in 0.5 M HCl with the presence of various concentrations of AEO7 corrosion inhibitors.

*C*_inh_ µmol L^−1^	*E*_a_ (kJ mol^−1^)	ΔH^*^ (kJ Mol^−1^)	ΔS^*^ (J mol^−1^ K^−1^)
0	35.70	−33.12	−77.96
20	39.92	−37.31	−73.36
30	42.13	−38.39	−68.94
35	47.05	−44.43	−57.08
40	49.60	−46.40	−54.36

A similar interpretation can be made using the thermodynamic equation below^30,84^:

21 $$RT=Ea-{\rm{\Delta }}{H}^{\ast }$$

The values of ΔH* are smaller compared to those of *E*a. The ΔS* values are large and negative. The ΔS* value becomes less negative with increasing AEO7 concentration indicating a decline in the level of perturbation of the AEO7 molecule to move from the reactant state to the activated complex one^82^.

### Microscope Analysis

**Figure 11:**
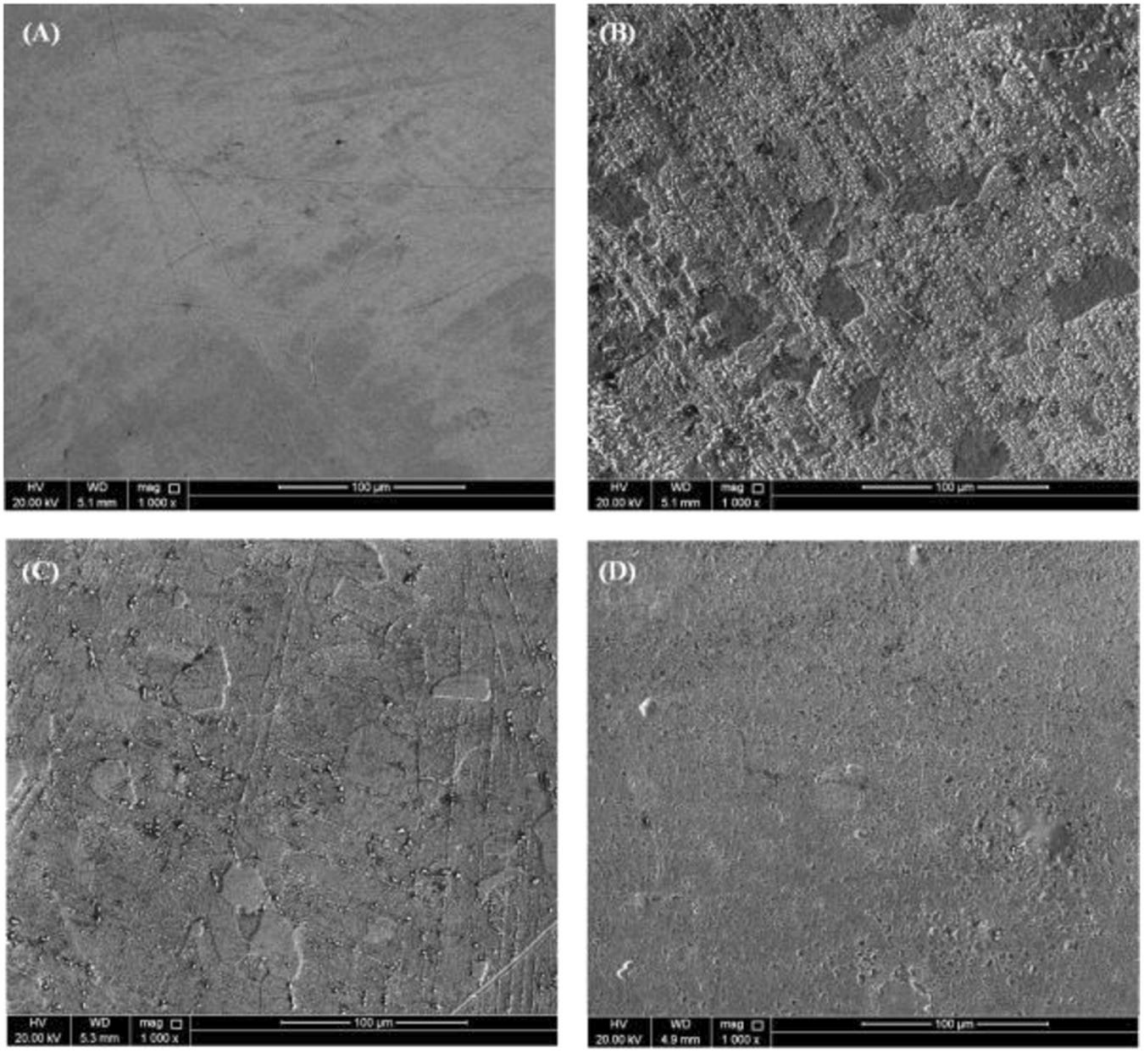
SEM micrographs for (**A**) a polished C-steel coupon in the (**B**) absence and (**C**) presence of 40 µmol L^−1^ of AEO7 alone and (**D**) in the presence of 1 × 10^−3^ M of KI along with 40 µmol L^−1^ of AEO7 in 0.5 M HCl medium.

Figures 11 and S2 compare the surface morphology of (A) a polished C-steel coupon using the SEM (Fig. 11) and optical profilometry (Figure S2) with another coupon after immersion for 24 h in 0.5 M HCl in the (B) absence and (C) presence of 40 µmol L^−1^ of AEO7 alone and (D) in presence of 1 × 10^−3^ M of KI along with 40 µmol L^−1^ of AEO7. The polished coupons did not exhibit any noticeable defects, except polishing scratches (Fig. 11A) with a surface roughness (*R*_a_ = 0.02 μm). After immersion in 0.5 M HCl without any inhibitor, heavy corrosion attack takes place (Fig. 11B) and *R*_a_ increases to 3.2 μm. In Figs 11C and 13C, where the AEO7 corrosion inhibitor exists, the corrosion is decreased significantly compared with that in its absence (Figs 11B and S2B) and *R*a decreases to 0.12 μm. In presence of 1 × 10^−3^ M KI in addition to 40 µmol L^−1^ of AEO7, a synergistic effect take place as the surface look clean and *R*_a_ decreases significantly to 0.06 μm.

**Figure 12:**
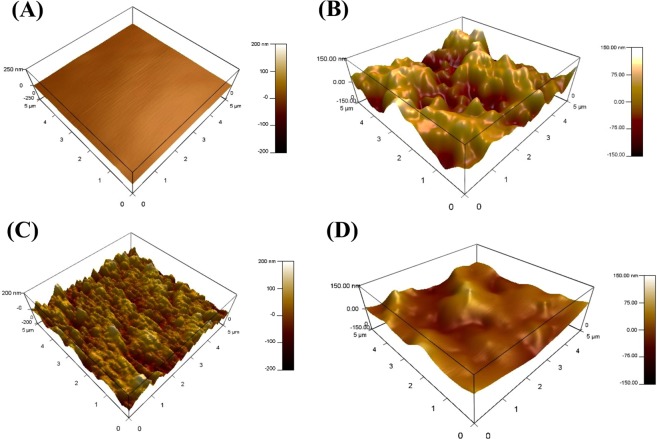
AFM images of (**A**) C-steel after polishing, in (**B**) absence, (**C**) presence of 40 µmol. L^−1^ of AEO7 corrosion inhibitor only and (**D**) in the presence of 1 × 10^−3^ M KI along with 40 µmol L^−1^ of AEO7 in 0.5 M HCl medium.

**Figure 13:**
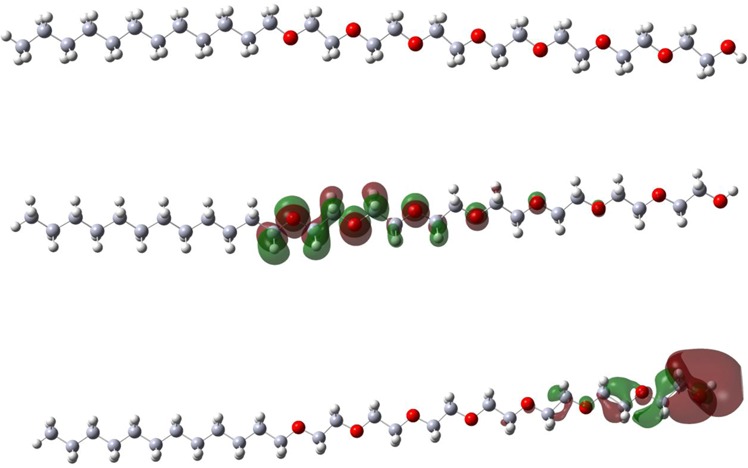
The optimized molecular structure of the AEO7 at the B3LYP/cc-pvdz level of theory (upper) and its frontier molecular orbital density distributions: (middle) HOMO and (lower) LUMO.

### AFM analysis

The three-dimensional AFM technique is one of the ideal approaches for exploring the surface topography at the nano and microlevels. It is also a powerful method for elucidating the efficiency of the corrosion inhibitor^84^. The optical profilometry provides qualitative images at the microscale level. However, the quantitative details for the surface roughness of C-steel can be determined by AFM where the mean roughness factor (*R*_a_) can be measured. The *R*_a_ values show that the roughness of C-steel immersed in 0.5 M HCl increases from 20 nm for the unexposed specimen to 320 nm for the coupon corroded in an uninhibited-HCl solution. However, the addition of 40 µmol L^−1^ of AEO7 reduces the *R*_a_ to 120 nm due to the formed adsorbed protective layer. Moreover, upon adding 1 × 10^−3^ M KI to the AEO7, the *R*_a_ is reduced to 65 nm. These results are clear in the AFM micrographs shown in Fig. 12. Both SEM and AFM measurements confirm the high corrosion *IE*% of AEO7 especially in the presence of KI.

### Quantum Chemical Calculations

Figure 13 represents the optimized structure of the inhibitor as well as the corresponding frontier molecular orbitals namely HOMO and LUMO. The molecule consists of a polar part composed of alternative eight oxygen atoms and ethylene groups linked to a zigzag non-polar hydrocarbon. The population of the electron density is focused on the oxygen atoms suggesting that heteroatoms behave as active sites with the highest ability to interact with the metal surface. The presence of these electronegative atoms increases the polarity of the molecule as well as their interaction with the charged sites on the metal surface enhancing the adsorption probability and then preventing interaction of the surface with the environment^85,86^.

**Figure 14:**
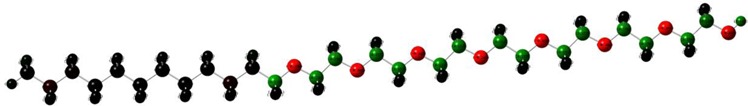
Mulliken charges of the AEO7 calculated at the B3LYP/cc-pvdz level of theory.

Figure 14 depicts the Mulliken charge distributions of AEO7. The highest electron densities are located at oxygen atoms (≈−0.34 au) indicating that oxygen atoms are the active centers, which have the strongest ability to interact of the metal surface. This finding is in line with the HOMO populations. This interaction with the metal surface with several numbers of active centers provides a good protective layer on the metal surface.

The quantum chemical parameters are calculated and listed in Table 8. The large energy gap decreases the ability of the inhibitor to form coordinate bonds with the *d*-orbital of the metal by donating and accepting electrons. Moreover, large global hardness and small global softness values reveal the inhibitor resistance to charge transfer and decreased the ability to receive electrons. However, the polarity of the molecule as inferred from the value of the dipole moment suggests a good electrostatic interaction with the metal surface and in turn enhances the inhibition ability. Therefore, these results are in good agreement with the experimental data i.e. the inhibitor favors the physical adsorption on the metal surface^87^.

**Table 8:** Quantum chemical parameters of AEO7 calculated at the B3LYP/cc-pvdz level of theory

∆*E*_gap_(eV)	*I* (eV)	*A* (eV)	γ (eV)	*X* (eV)	*δ* (eV)	*μ* (D)
8.21	6.89	−1.31	4.10	2.79	0.24	0.48

## Conclusion

AEO7 is an effective corrosion inhibitor for C-steel in 0.5 M HCl solutions. The presence of KI synergistically enhanced the corrosion *IE*% and the adsorption of AEO7 at the C-steel surface at ambient temperature. Weight loss results reveal a high corrosion *IE*% especially at room temperatures. EIS and polarization measurements confirm the adsorption of AEO7 corrosion inhibitor molecules on the C-steel surface and the formation of a protective layer that suppresses the C-steel oxidation and the hydrogen ion reduction reactions at the anodic and cathodic sites, respectively. AEO7 molecules are exothermically physisorbed on the C-steel surface, and the adsorption follows the Freundlich isotherm. SEM, optical profilometry and AFM measurements reveal a smooth surface of the C-steel coupon when immersed in an AEO7-inhibited HCl solution compared to the cases when an uninhibited HCl solution is used where severe corrosion takes place, which increases the surface roughness significantly.

## Supplementary information

AEO7 Surfactant as an Eco-Friendly Corrosion Inhibitor for Carbon Steel in HCl solution

## Data Availability

The raw data required to reproduce these findings can be shared at any time based on direct requests to the authors.
