# Supplementary material for: AEO7 Surfactant as an Eco-Friendly Corrosion Inhibitor for Carbon Steel in HCl solution
Source: Sci Rep. 2019 Feb 19;9:2319. doi: 10.1038/s41598-018-37254-7 (PMC6381149; doi:10.1038/s41598-018-37254-7)
Supplement: Supplementary file 1 — AEO7 Surfactant as an Eco-Friendly Corrosion Inhibitor for Carbon Steel in HCl solution [file 41598_2018_37254_MOESM1_ESM.docx]

**Supplementary Information**

**AEO7 Surfactant as an Eco-Friendly Corrosion Inhibitor for Carbon Steel in HCl solution**

Mostafa H. Sliem^1^, Mohamed Afifi^2^, Ahmed Bahgat Radwan^1^, Eman M. Fayyad^1, 3^, Mohamed F. Shibl^2, 4,*^, Fakiha E. Heakal^2^ and Aboubakr M. Abdullah^1,*^

^1^ Center for Advanced Materials, Qatar University, Doha, P.O. Box 2713, Qatar.

^2^ Chemistry Department, Faculty of Science, Cairo University, Giza 12613, Egypt.

^3^ Physical Chemistry Department, National Research Center, Dokki, Cairo, Egypt.

^4^ Gas Processing Center, Faculty of Engineering, Qatar University, Doha P.O. Box 2713, Qatar.

**Figure S1.** The measured EIS Bode and phase angle plots (dotted) and their fitted curves (solid lines) for C-steel in 0.5 M HCl in the presence of 0, 20, 30, 35 and 40 µmol L^-1^ of AEO7 corrosion inhibitor at (A) 20, (B) 30, (C) 40 and (D) 50 °C within the frequency range of 0.1 Hz to 100 kHz at OCP. The EIS is fitted using the same one-time constant electrical equivalent circuit shown in Figure 5.

**Figure S2:** Optical profilometry images for (A) a polished C-steel coupon in the (B) absence and (C) presence of 40 µmol L^-1^ of AEO7 alone and (D) in presence of 1x10^-3^ M of KI along with 40 µmol L^-1^ of AEO7.

**Table S1**: The electrochemical parameters and corrosion inhibition efficiencies obtained from the measured impedance spectra of C-steel in 0.5 M HCl at different concentrations of KI at room temperature.

| *C*_inh,_ mol L^-1^ | *R*_ct,,_ Ω cm^2^ | *CPE* | | | $\theta$ | *Corrosion IE*% |
| --- | --- | --- | --- | --- | --- | --- |
|  |  | *Y*_0_ ×10^-6^ s^n^ ohm^−1^ cm^−2^ | *C_dl_,* µF | *n* |  |  |
| Blank | 43.1 | 257.0 | 119.7 | 0.855 | ----- | ----- |
| 1x10-3 | 79 | 91.38 | 55.77 | 0.909 | 0.45 | 45.2 |
| 2.5x10-3 | 112 | 83.72 | 65.47 | 0.950 | 0.61 | 61.1 |
| 5x10-3 | 162 | 71.76 | 36.68 | 0.866 | 0.73 | 73.4 |
| 7.5x10-3 | 229 | 49.32 | 27.22 | 0.883 | 0.81 | 81.0 |
| 1x10-2 | 310 | 41.37 | 18.37 | 0.843 | 0.86 | 86.5 |
